# Melatonin modulates baroreflex control via area postrema

**DOI:** 10.1002/brb3.123

**Published:** 2013-02-17

**Authors:** Luciana A Campos, Jose Cipolla-Neto, Lisete C Michelini

**Affiliations:** 1 São José dos Campos Technology Park, University Camilo Castelo Branco (UNICASTELO)São Paulo, Brazil; 2 Department of Physiology Institute of Biomedical Sciences, University of Sao PauloSao Paulo, Brazil

**Keywords:** Area postrema, baroreflex, blood pressure, heart rate, melatonin

## Abstract

Pineal gland and its hormone melatonin have been implicated in modulation of cardiovascular system. We aimed at studying the effects of melatonin on baroreflex sensitivity and the role of area postrema, as a component modulator of baroreflex arch. Mean arterial pressure (MAP) and heart rate (HR) were recorded in conscious freely moving rats. Baroreceptor reflex sensitivity was assessed by determining the HR responses to ramped infusions of phenylephrine (PE) and sodium nitroprusside (SNP)-induced MAP changes. Melatonin bolus (0.11 mg/kg) immediately followed by its continuous infusion (0.43 × 10^−9^ mol/L at a rate of 0.65 mL/h for 30 min) in healthy normotensive rats produced a downward shift of baroreceptor reflex control with a substantial inhibition of reflex tachycardia (−32%) and potentiation of reflex bradycardia (+20%). Ablation of area postrema (APX group) induced a sustained decrease of MAP (101 ± 3 vs. 116 ± 3 mmHg, *P* < 0.05 in comparison with sham rats, respectively). The melatonin-induced alterations of baroreflex function observed in the sham group were abolished in the APX group. We conclude that circulating melatonin can modulate baroreceptor reflex control of HR, thus resetting it toward lower HR values. The modulatory effects of melatonin may be mediated via melatonin receptors in the area postrema, located outside the blood–brain barrier.

## Introduction

Melatonin (*N*-acetyl-5methoxytryptamine), a hormone produced and released by the pineal gland in a circadian pattern with high plasma levels at night (Reiter 1993), has been implicated in several physiological processes, such as modulation of biological rhythms (Pevet 2000), neuro-immune axis (Martins et al. 1998; Reiter and Maestroni 1999), glucose tolerance and insulin action (Lima et al. 1998), and reproductive activity (Luboshitzky and Lavie 1999). Various studies have indicated that a decrease in melatonin levels may be associated with cardiovascular diseases such as hypertension (Zanoboni and Zanoboni-Muciaccia 1967; Zanoboni et al. 1978; Jonas et al. 2003; Paulis and Simko 2007). Melatonin administration can reverse a transient pinealectomy-induced hypertension (Zanoboni and Zanoboni-Muciaccia 1967; Holmes and Sugden 1976; Zanoboni et al. 1978; Simko and Paulis 2007). There is evidence that exogenous melatonin may decrease mean arterial pressure (MAP) and heart rate (HR) in both normotensives and hypertensive rats and humans (Kawashima et al. 1984; Mallion et al. 1990; Cagnacci et al. 2005), and that the experimental abolition of nocturnal melatonin release (Brown et al. 1991) results in suppression of circadian blood pressure variability (Briaud et al. 2004). Another finding indicated that subchronic per os treatment with melatonin may increase cardiac baroreflex in spontaneously hypertensive rats (SHR) (Girouard et al. 2004). In this study, we sought to investigate the effects of acute infusion of melatonin on baroreflex control and the possible mechanisms involved in these effects. A strong candidate for signaling melatonin levels in orderto trigger baroreflex modulation is the area postrema (AP), a circumventricular organ in the dorsal brainstem closely related to cardiovascular controlling areas that contain high density of melatonin receptors MT1 and MT2 (Williams et al. 1995). Besides, it is known that area postrema densely projects to the nucleus of tractus solitarii (NTS), an important integrative area for baroreflex control (Shapiro and Miselis 1985; Johnson and Gross 1993). Thus, melatonin released into the plasma may act on area postrema, which is devoid of blood–brain barrier (BBB) and that has a high density of melatonin receptors, being thus a “window” to detect melatonin's plasma levels. Therefore, we sought to investigate the role of area postrema on the possible melatonin-induced effects on baroreflex control.

## Material and Methods

Male Wistar-Kyoto (WKY) rats, 3 months of age, were obtained from the animal facilities of the Biomedical Sciences Institute – Department of Physiology and Biophysics, University of Sao Paulo, Brazil. The rats were housed individually in a synchronized 12-h light–dark cycle (light: 6 am to 6 pm, 200 lux; dark 6 pm to 6 am, <0.1 lux), and temperature controled room (22 ± 2°C) at least 2 weeks prior to the experiments. A standard rat diet and tap water were supplied ad libitum. All experimental protocols were performed in accordance with the ethical principles in animal research of the Brazilian College of Animal Experimentation, guidelines for the human use of laboratory animals by the State of Sao Paulo and approved by the Ethical Committee of the Biomedical Sciences Institute of the University of Sao Paulo.

### Measurements of cardiovascular parameters

For blood pressure and HR recordings, catheters were implanted into the left femoral artery, and for drug administration, catheters were placed into the left femoral vein under anesthesia with ketamine–xylazine (70:6 mg/kg im). The catheter was tunneled subcutaneously and attached to the back muscles of the neck. Catheters were implanted 24 h before the experiments to allow a complete recovery from anesthesia. Arterial pressure and HR were recorded by connecting the arterial catheter to a flow-through pressure transducer (P23XL, Gould, Cleveland, OH), which was then connected to a recording system (carrier amplifier + Biotach, RS 3400 recorder Gould). The rat was allowed to rest for stabilization of cardiovascular parameters.

### Evaluation of baroreflex bradycardia and tachycardia

Arterial baroreceptors were stimulated by a series of increasing doses of intravenous injections of phenylephrine (PE) and sodium nitroprusside (SNP). Response logistic function curves of MAP and HR were obtained. The baseline values and peak changes of MAP and HR were analyzed. The reflex test with progressive doses of PE and SNP lasted for about 30–40 min. MAP and HR were recorded continuously and the mean baseline values of blood pressure and HR (between the responses obtained to different doses) used for plotting the midpoint of the curves. PE injections (0.1, 0.2, 0.4, 0.8, 1.6, 3.2, 6.4, 12.8 μg/kg) and SNP (0.2, 0.4, 0.8, 1.6, 3.2, 6.4, 12.8, 25.6 μg/kg) were randomized. Also, melatonin infusions were randomized.

### Melatonin administration

Both baseline values and responses to load/unload of baroreceptors with bolus of PE and SNP, respectively, were recorded during continuous intravenous infusion of either vehicle (10^−7^ V:V of alcohol in saline 0.9%, at a rate of 0.65 mL/h) or of melatonin (0.43 × 10^−9^ mol/L, at a rate of 0.65 mL/h) for 30 min, which was light protected throughout the experiment. To the group receiving melatonin, it was first given a bolus injection of melatonin (0.11 mL), which was immediately followed by its continuous infusion. The dose was chosen to mimic physiological concentration of plasma melatonin (Esteban et al. 2004). The aimed final plasmatic dosage was 100 pg/mL that is the mean daily peak of melatonin in rats. In a pilot experiment, the final dosage was 105 ± 34 pg/mL (*n* = 6). All melatonin infusion experiments started at 9 am, when melatonin levels are minimal.

### Area postrema ablation

Rats were anesthetized with Hypnol 30% (0.15 mL/kg ip) and placed on a stereotaxic apparatus (David Kopf Instruments, CA). A midline incision was made in the dorsum of the neck, and muscles were separated to expose the foramen magnum. The atlanto-occipital membrane was opened to expose the obex and the area postrema. In the group submitted to area postrema ablation (APX group), an electrode (stainless steel, insulated with epoxyde, except for the tip) was guided stereotaxically under direct vision into the area postrema, and inserted 0.5 mm below the brain stem surface. An anodal direct current of 1 mA was passed for 8 sec (DC LM5 Lesion Maker, Grass Instruments Co., Quincy, MA), with the cathode attached to the skin of the neck (APX group, *n* = 6). As control, a sham group (*n* = 6) was used, where the obex was surgically exposed, but otherwise left untouched. Immediately after the surgical procedure, the muscles and skin were sutured, and Penicillin G (30,000 U) was administered intramuscularly. The rats were allowed to recover from the operation for a period of 4–5 days before the experiments. The experimental protocols were performed in conscious and unrestrained animals. Baroreflex was evaluated after vehicle and melatonin infusion in each APX or sham rat.

### Histology

**Figure 1:**
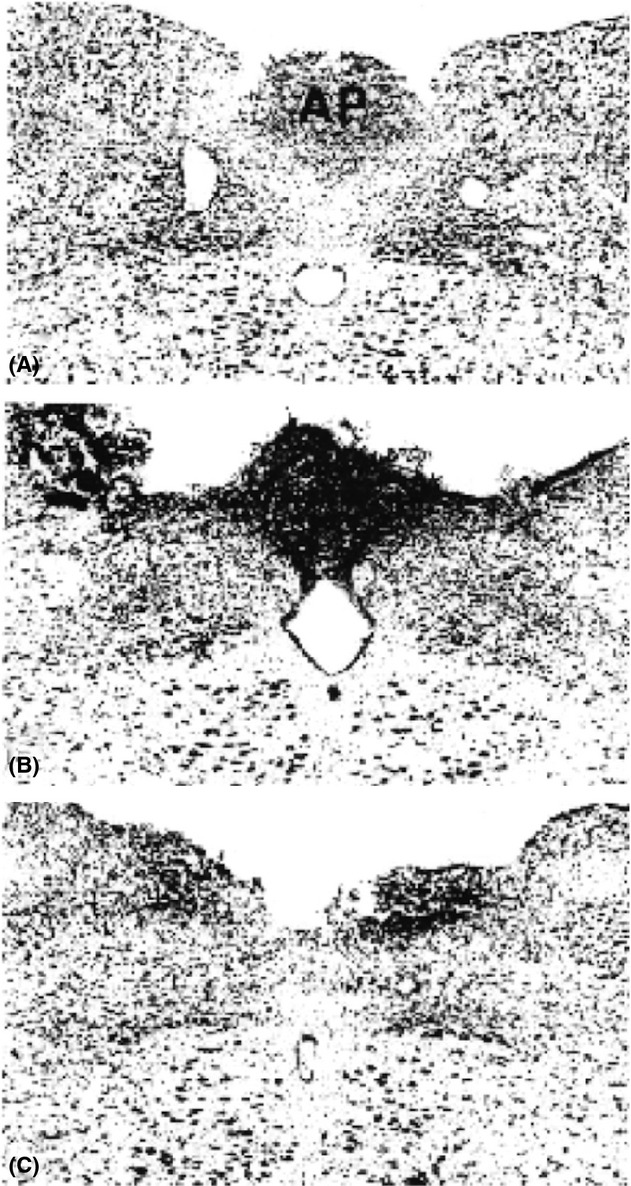
Photomicrographs showing histological sections of an intact area postrema (A) and of area postrema electrolytically lesioned (B and C). Figure 1B and C characterize two types of lesions obtained: cell death (black cells) and their complete removal, respectively.

At the end of the experimental protocol, the rats were deeply anesthetized (Nembutal, 50 mg/kg) and perfused transcardially with 30–40 mL of saline followed by 10% buffered formalin. The brains were removed and stored in 10% formalin for 1 week, and postfixed (48 h) in 10% sucrose formalin before sectioning. The medulla oblongata was cut into 40-μm serial coronal frozen sections with a cryostat (Jung – SM2000R). Sections were Nissl stained and examined by light microscopy to determine the location and extent of lesion according to the Atlas of Paxinos and Watson (Paxinos et al. 1985). Only data from rats with a complete and restricted area postrema ablation were considered in the APX-lesioned group (Fig. 1).

### Data analysis and statistics

Sigmoidal logistic equation was used (Kent et al. 1972; Head and McCarty 1987; Pontieri et al. 1998) to analyze baroreceptor reflex, which correlated absolute HR and MAP values. The applied equation was:


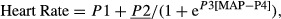


where *P*1 = lower HR plateau, *P*2 = HR range, *P*3 = curvature coefficient, which is independent of the range, and *P*4 = MAP_50%_, that is, MAP at half the HR range. The average gain (*G*) or slope of the curve between the two inflection points was given by *G* = −*P*2 × *P*3/4. The upper plateau was calculated as *P*1 + HR range (*P*2).

The baseline values of MAP and HR, maximal pressor and depressor responses to PE and SNP, and the parameters of both linear fit and sigmoidal fitting of sham and APX groups infused with either vehicle or melatonin were analyzed by two-way analysis of variance with repeated measures (vehicle vs. melatonin infusion in each group with or without area postrema ablation). Student-Newman–Keuls was used as a post hoc test. Data are expressed as means ± SE. *P* < 0.05 was regarded as significantly different.

## Results

### Melatonin infusion decreases arterial pressure and HR

**Figure 2:**
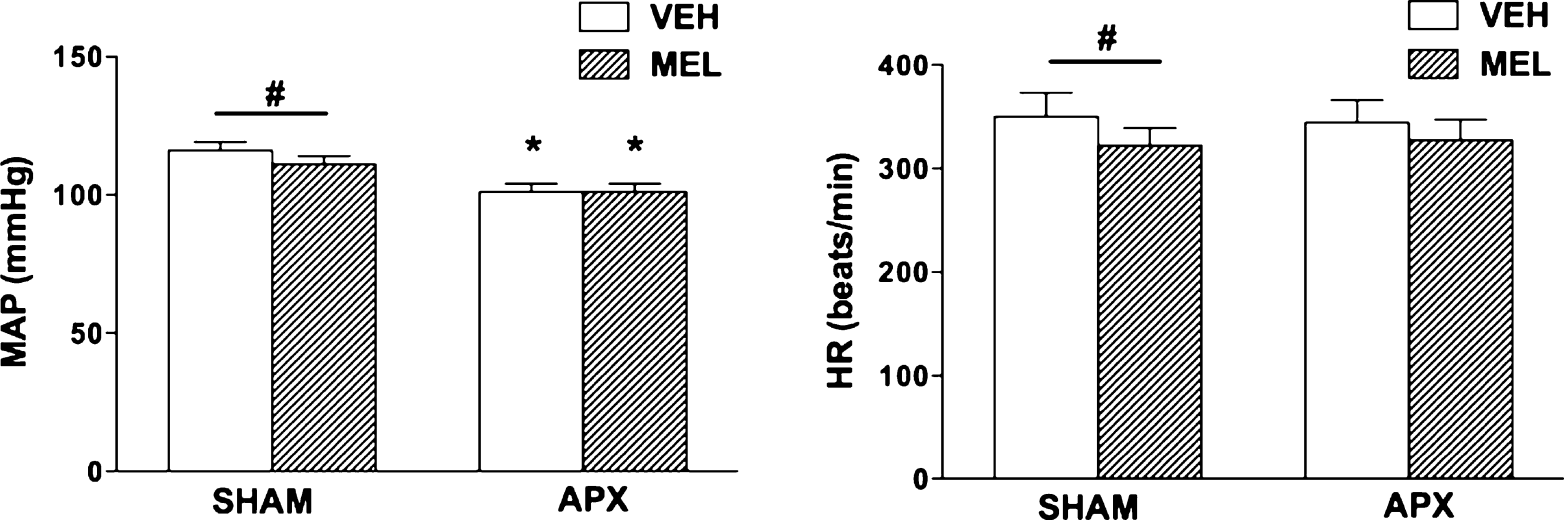
Avarage values of mean arterial pressure (MAP) and heart rate (HR) in sham-operated (*n* = 6) and area postrema-ablated group (APX, *n* = 6). Measurements were made during intravenous infusions of vehicle (VEH) and melatonin (MEL) in conscious rats. Significances (*P* < 0.05): * versus sham group, # versus VEH.

In control normotensive rats, melatonin infusion induced an immediate and stable 4.3% reduction of MAP (116 ± 3 vs. 111 ± 3 mmHg, *P* < 0.05, Fig. 2) and an 8% reduction of HR (350 ± 23 vs. 322 ± 17 beats/min, *P* < 0.05, Fig. 2). Levels of MAP and HR returned to normal after the end of melatonin infusion.

### Reduced arterial pressure in rats with ablated area postrema

Rats submitted to APX (Fig. 1), the vehicle-treated group, exhibited a significant decrease in basal MAP compared with vehicle-treated sham-operated controls (101 ± 3 vs. 116 ± 3 mmHg, *P* < 0.05, Fig. 2), with no basal HR changes (344 ± 22 vs. 350 ± 23 beats/min, APX vehicle treated vs. sham vehicle treated, respectively, Fig. 2).

### Acute melatonin infusion resets the baroreflex

**Figure 3:**
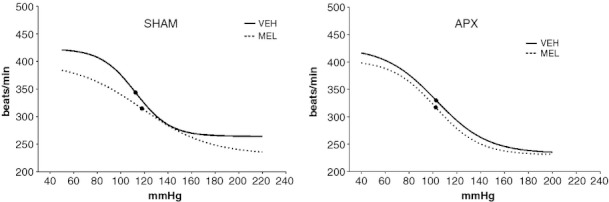
Average of logistic function curve with the relation between mean arterial pressure (MAP) and heart rate (HR) during intravenous administration of vehicle (VEH) or melatonin (MEL) in conscious sham-operated animals and area postrema (APX)-ablated group. Significances are shown in Table 1.

The changes in baroreceptor reflex sensitivity during melatonin infusion were assessed by means of a sigmoidal curve-fitting analysis. A clear upper and lower plateau (reflex tachycardia and bradycardia, respectively) was noted in both sham and area postrema-ablated (APX) groups. Acute continuous melatonin infusion in the sham-operated group (Fig. 3) determined significant downward displacement of HR responses elicited by PE and SNP (lower plateau: 231 ± 19 vs. 264 ± 20 beats/min, *P* < 0.05, and upper plateau: 398 ± 12 vs. 423 ± 14 beats/min, *P* < 0.05, melatonin vs. vehicle, respectively, Fig. 3), with no significant change in the range (167 ± 10 vs. 159 ± 9 beats/min) or sensitivity (gain: −1.48 ± 0.68 vs. −2.74 ± 0.71 beats/min per mmHg, Table 1) of the reflex. Linear regression analysis showed that melatonin administration caused a 24% increase in bradycardic responses to PE (−1.82 ± 0.22 vs. −1.46 ± 0.17 beats/min per mmHg, Table 1) and a 32% decrease in tachycardic responses to SNP (−2.71 ± 0.44 vs. −4.00 ± 0.61 beats/min per mmHg, Table 1).

**Table 1:** Values of the mean arterial pressure (MAP), heart rate (HR), parameters of logistic function curve fitting of baroreceptor reflex control of heart rate in conscious sham and area postrema lesion (APX-group) animals administered intravenously with vehicle (VEH) or melatonin (MEL)

	sham group		APX group	
		
	Vehicle	Melatonin	Vehicle	Melatonin
Logistic function curve fit				
Lower plateau (beats/min)	264 ± 20	231 ± 19^2^	233 ± 9	230 ± 9
Upper plateau (beats/min)	423 ± 14	398 ± 12^2^	426 ± 13	404 ± 16
Range (beats/min)	159 ± 9	167 ± 10	193 ± 8	173 ± 11
MAP_50_ (mmHg)	113 ± 4	118 ± 3	103 ± 5^1^	102 ± 5^1^
Gain (beats/min per mmHg)	−2.74 ± 0.71	−1.48 ± 0.68	−2.23 ± 0.22	−2.29 ± 0.41

Values are mean ± SEM. MAP_50_ = mean arterial pressure at midrange. Significances (*P* < 0.05): ^1^versus sham group, ^2^versus VEH.

### Melatonin-induced alteration of baroreflex is abolished by ablation of area postrema

The reflex HR responses, which were elicited by alternate intravenous bolus injections of PE (delta +5 to +60 mmHg) and SNP (delta −5 to −24 mmHg) were similar in both sham and APX groups. In the APX group (Fig. 3), continuous melatonin infusion did not alter the HR responses elicited by PE and SNP (lower plateau: 230 ± 9 vs. 233 ± 9 beats/min, and upper plateau: 404 ± 16 vs. 426 ± 13 beats/min, melatonin vs. vehicle, respectively, Fig. 3). Also, there was no significant change in the range (173 ± 11 vs. 193 ± 8 beats/min, Table 1) or sensitivity (gain: −2.29 ± 0.41 vs. 2.23 ± 0.22 beats/min per mmHg, Table 1) of the reflex. In agreement with the baseline MAP decrease, MAP_50%_ was reduced in the APX group in comparison with sham group (103 ± 5 vs. 113 ± 4 mmHg, Table 1). After area postrema lesion, melatonin infusion was ineffective to alter baroreflex control of HR (Fig. 3, Table 1).

## Discussion

The presented data support the central effects of melatonin as they report reduction of both blood pressure and HR after melatonin infusion. We showed that circulating melatonin, acting through the area postrema, reduces baseline pressure and HR and resets baroreceptor reflex control toward lower HR values. On the other hand, ablation of area postrema abolishes melatonin effects on baroreflex and decreases arterial pressure.

Pineal gland and its hormone melatonin are well known for modulating circadian biological rhythms. Melatonin is secreted by pineal gland during the dark period of the day to modulate biological activity of various organs and system through G-protein-coupled membrane-bound melatonin receptors. A direct effect of melatonin on blood pressure has been described. Continuous melatonin infusion was effective to reduce blood pressure of hypertensive rats (Kawashima et al. 1984) and hypertensive and normotensive humans (Cagnacci et al. 2005; Simko and Paulis 2007; Grossman et al. 2011). Moreover, an improvement of baroreflex by long-term melatonin treatment in hypertensive rats SHR has been reported (Girouard et al. 2004). Our results indicate that acute infusion of melatonin may reduce blood pressure and HR levels also in normotensive rats. Melatonin receptors are expressed in cardiovascular system (Peliciari-Garcia et al. 2011; Schepelmann et al. 2011) and also in several brain nuclei including area postrema (Weaver et al. 1989; Williams 1989), which densely projects to the NTS, an important integrative area for baroreflex control (Michelini 2007). The existence of high-density melatonin receptors in area postrema together with our data suggest a role for melatonin in baroreflex function of this nucleus.

It is well known that area postrema has anatomical connections with important cardiovascular areas in the brain. The area postrema receives afferent input and sends extensive efferent projections to autonomic control centers in the medulla, pons, and forebrain (van der Kooy and Koda 1983; Dampney 1994). Moreover, there are many contingents of efferent projections from the area postrema to the NTS, dorsal motor nucleus of the vagus, and lateral parabrachial nucleus of the pons (van der Kooy and Koda 1983; Shapiro and Miselis 1985). In this study, area postrema ablation per se did not affect baroreflex function, indicating that neurons within the area postrema are not part of the reflex arc. However, area postrema ablation abolished the melatonin-induced downward resetting of the reflex confirming a modulatory effect. Our results suggest that melatonin changes the operating set point of the arterial baroreflex through an area postrema-mediated mechanism. This effect, naturally occuring during the night, might contribute not only to nocturnal pressure fall exhibited by dipper individuals (White 1999a,b; Verdecchia 2000), but also to the simultaneous baroreceptor resetting. Area postrema lesions may lead to anorexia-induced loss of body weight (Kenney et al. 1994). A significant decrease in body weight per se could alter baseline cardiovascular parameters. However, in our study, APX-induced body weight decrease was only 11% and did not reach a significant difference from sham. Besides, other reports showed that blood pressure was not affected by the decrease in body weight, at least in the time frame of our experimental protocol (Collister and Osborn 1998; Curtis et al. 2003). This is why we consider that the blood pressure decrease observed in APX rats was not due to a decrease in body weight.

The hypotensive action of melatonin appears to be associated with an inhibition of basal sympathoadrenal tone in SHR and WKY rats (K-Laflamme et al. 1998). It has been proposed that hypertension may be the result of melatonin-induced epigenetic modifications in neurons of area postrema (Irmak and Sizlan 2006), which in turn may play a role in setting the arterial pressure to a higher operating set-point seen in hypertension (Joy and Lowe 1970; Fink et al. 1987; Wilson and Bonham 1994). These data corroborated with our results are suggesting that circulating melatonin released by the pineal during the night could contribute to reducing energetic cost (smaller pressure and HR with reset HR control), without changing the efficiency of the reflex control of HR.

It has been previously reported by another study that there is an improvement of baroreflex control by long-term melatonin treatment in hypertensive rats SHR (Girouard et al. 2004). A limitation of our study is that we did not investigate long-term effects of melatonin on area postrema ablation. The new finding of this study demonstrating a functional role of melatonin on the modulation of the baroreflex control possibly acting through its receptors in area postrema could be a first step for further studies on long-term effects of melatonin acting on area postrema with an impact on cardiovascular diseases.
